# Studies on APP metabolism related to age-associated mitochondrial dysfunction in APP/PS1 transgenic mice

**DOI:** 10.18632/aging.102451

**Published:** 2019-11-19

**Authors:** Lizhi Chen, Shicheng Xu, Tong Wu, Yijia Shao, Li Luo, Lingqi Zhou, Shanshan Ou, Hai Tang, Wenhua Huang, Kaihua Guo, Jie Xu

**Affiliations:** 1 Department of Clinical Anatomy, School of Basic Medical Sciences, Southern Medical University, Guangzhou, China; 2 Department of Ultrasound, The Third Affiliated Hospital of Sun Yat-Sen University, Guangzhou, China; 3 Department of Anatomy and Neurobiology, Zhongshan School of Medicine, Sun Yat-Sen University, Guangzhou, China; 4 Department of Anatomy, School of Basic Medicine, Guangdong Pharmaceutical University, Guangzhou, China; 5 Department of Anatomy, Guangdong Jiangmen Chinese Traditional Medicine College, Jiangmen, China

**Keywords:** mitochondria dysfunction, adenosine 5’-triphosphate, Amyloid-beta, platelets, APP/PS1 mice

## Abstract

The aging brain with mitochondrial dysfunction and a reduced adenosine 5’-triphosphate (ATP) has been implicated in the onset and progression of β-Amyloid (Aβ)-induced neuronal toxicity in AD. To unravel the function of ATP and the underlying mechanisms on AD development, APP/PS1 double transgenic mice and wild-type (WT) C57 mice at 6 and 10 months of age were studied. We demonstrated a decreased ATP release in the hippocampus and platelet of APP/PS1 mice, comparing to C57 mice at a relatively early age. Levels of Aβ were raised in both hippocampus and platelet of APP/PS1 mice, accompanied by a decrease of α-secretase activity and an increase of β-secretase activity. Moreover, our results presented an age-dependent rise in mitochondrial vulnerability to oxidation in APP/PS1 mice. In addition, we found decreased pSer473-Akt levels, increased GSK3β activity by inhibiting phosphorylation at Ser9 in aged APP/PS1 mice and these dysfunctions probably due to down-regulation of Bcl-2 and up-regulation of cleaved caspase-3. Therefore, we demonstrate that PI3K/Akt/GSK3β signaling pathway could be involved in Aβ-associated mitochondrial dysfunction of APP/PS1 mice and APP abnormal metabolism in platelet might provide potential biomarkers for early diagnosis of AD.

## INTRODUCTION

Alzheimer’s disease (AD) is a complicated and multifaceted neurodegenerative disease initiated by the misfolding and abnormal accumulation of intracellular and extracellular deposits [1]. Aggregation of toxic β-amyloid (Aβ) oligomers has been recognized as a prominent pathological hallmark for AD, and they are derived from the sequent proteolysis of membrane-bound amyloid precursor protein (APP) by β- and γ- secretases [2]. An increasing amount of evidence has shown that mitochondria is important intracellular organelle for Aβ accumulating [3, 4] and mitochondrial dysfunctions are heavily involved in AD [5]. Nevertheless, the causal link between mitochondrial dysfunction and AD pathogenesis is elusive.

Previous data indicate that AD-associated proteins and peptides are localized in mitochondria, which in part characterized by a decrease of ATP, impaired oxidative phosphorylation and increased reactive oxygen species (ROS) generation [6, 7]. Age-dependent Aβ deposition is proposed to have an interaction with mitochondrial bioenergetic homeostasis [8]. Mitochondria are particularly sensitive to the irreversible loss of neuronal function and the neuronal death that cause deficits in the activities of the respiratory chain complexes and ultimately results in an increase of free radical production and a decrease of adenosine 5'-triphosphate (ATP) production [9]. Mitochondria-derived ROS are sufficient to trigger an amyloidogenic process of APP both *in vitro* and *in vivo* [10]. Oxidative damage reflected by the oxidative modification of macromolecules in the organism occurs when pro-oxidant and free radicals are eliminated by endogenous antioxidative enzymes [11].

Mitochondria are the dominant location for ATP production via acetyl-coenzyme A (acetyl-CoA) oxidation and oxidative phosphorylation [12]. Platelet aggregation and activation are energy expenditure processes, which rely on metabolism of ATP and adenosine diphosphate (ADP) respectively [13]. The present study was designed to investigate the underlying molecular mechanisms for Aβ-induced mitochondrial dysfunction in hippocampi and platelets. We use the transgenic mice carrying APPswe/PS1dE9 mutations (APP/PS1 mice) as our AD model as they mimic critical AD symptoms such as robust Aβ accumulation and memory decline [14].

## RESULTS

### ATP production

**Figure 1:**
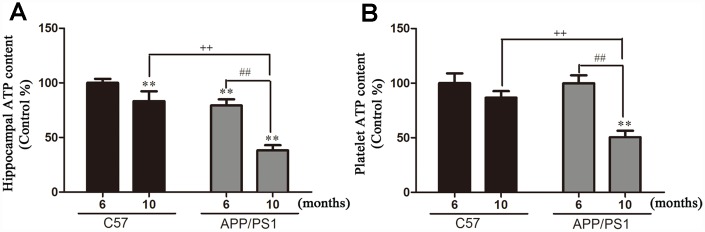
**Hippocampal and platelets ATP content in and from APP/PS1 transgenic mice and C57 mice.** (**A**) ATP content in hippocampi; (**B**) ATP content in platelets. Results are expressed as a mean ± SD value of six experiments measured in duplicate. The values are expressed as percentage of control, which is set to 100%. ^**^P < 0.01 *vs.* C57 (6 months), ^##^*P* < 0.01 *vs.* APP/PS1 (6 months), ^++^*P* < 0.01 *vs.* C57 (10 months).

ATP production in the hippocampi and platelets. As shown in Figure 1A, significantly decreased levels of ATP production were found in the hippocampi from the 6-month-old and 10-month-old APP/PS1 mice, relative to age-matched C57 mice. As shown in Figure 1B, ATP content in the platelet significantly decreased with age, and significantly increased compared with C57 group at 10 months, but the decrease was not significant at 6 months.

### Aβ accumulation

**Figure 2:**
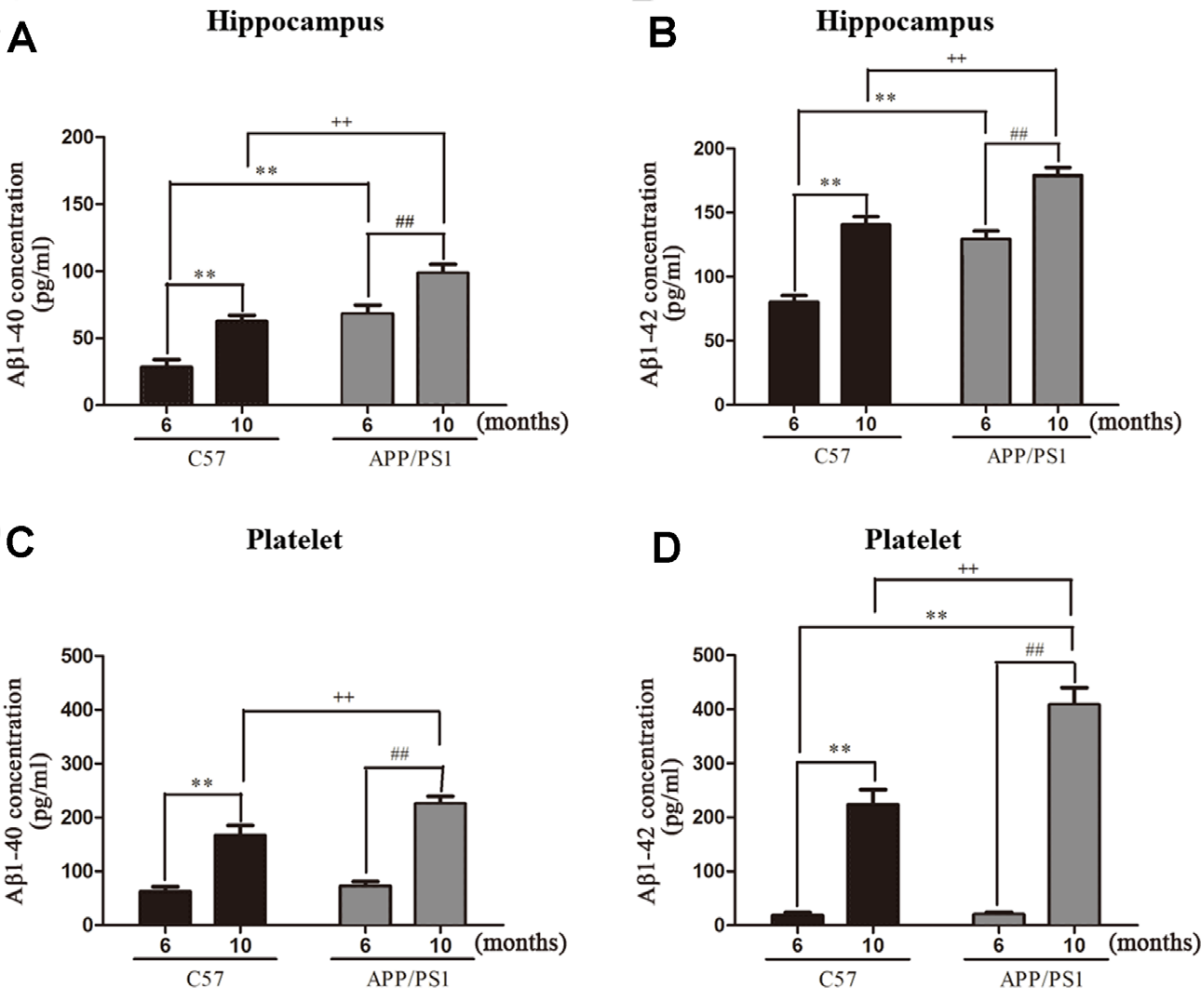
**Aβ1-40 and Aβ1-42 levels in hippocampus and platelets from APP/PS1 transgenic mice and C57 mice.** (**A**) Aβ1-40 levels in hippocampus; (**B**) Aβ1-42 levels in hippocampus; (**C**) Aβ1-40 levels in platelets; (**D**) Aβ1-42 levels in platelets. Values are expressed as mean ± SD of six experiments measured in duplicate. ^**^P < 0.01 *vs.* C57 (6 months), ^##^*P* < 0.01 *vs.* APP/PS1 (6 months), ^++^*P* < 0.01 *vs.* C57 (10 months).

**Figure 3:**
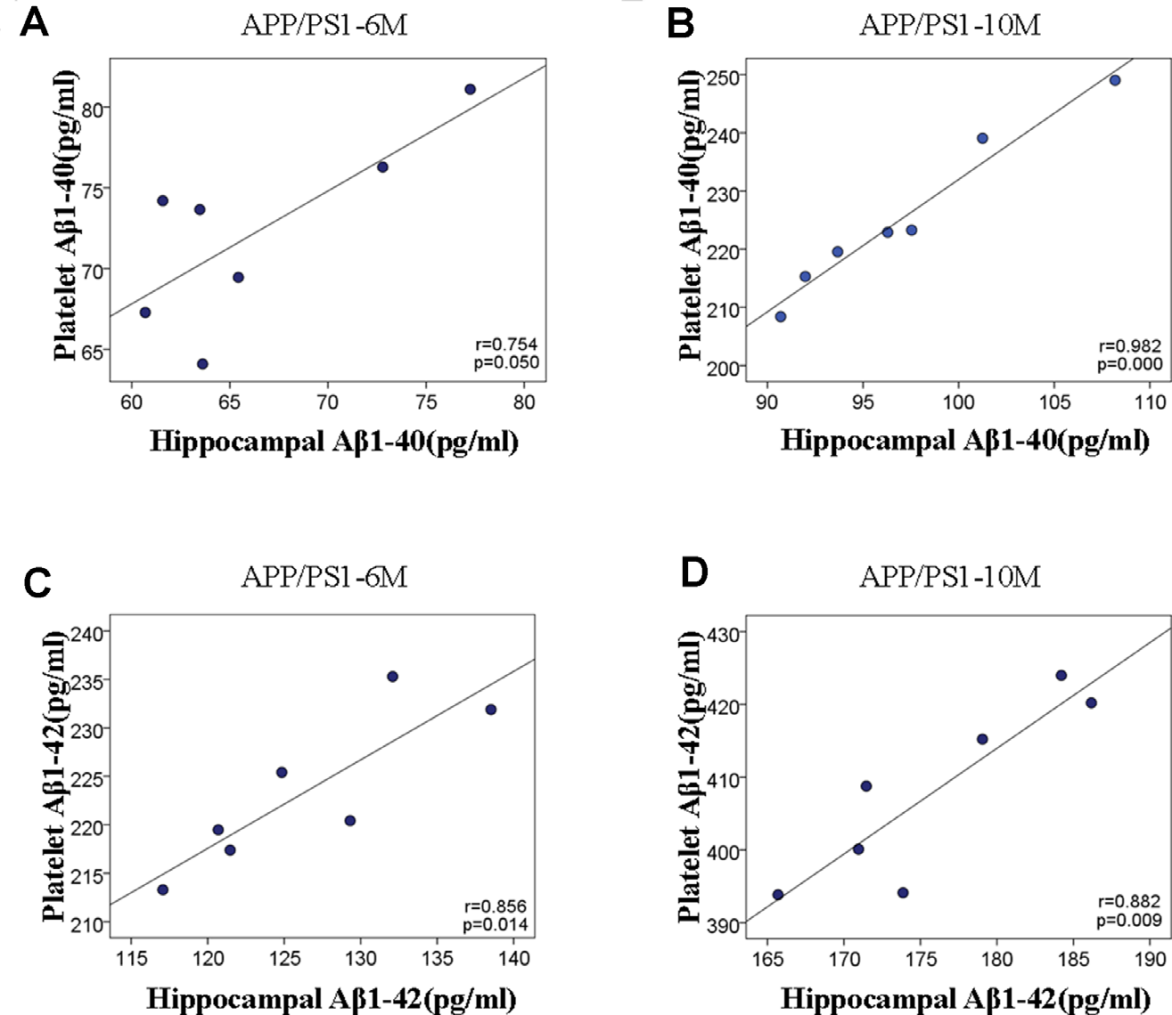
**Scatter plots of the correlation between hippocampal and platelet Aβ variables in APP/PS1 transgenic mice and C57 mice.** (**A**) The correlation analysis between hippocampal Aβ1-40 and platelet Aβ1-40 at 6 and 10 months. (**B**) The correlation analysis between hippocampal Aβ1-42 and platelet Aβ1-42 at 6 and 10months.

Aβ accumulation in the hippocampi and platelets. We detected the Aβ1-40 and Aβ1-42 protein levels in the hippocampi and platelets of APP/PS1 mice with the ELISA assay. As shown in Figure 2A and 2B, both Aβ1–40 and Aβ1–42 levels were significantly increased in the hippocampi of APP/PS1 mice compared with C57 mice. As expected, the accumulations of Aβ1-40 and Aβ1-42 in the platelets of APP/PS1 group are both significantly increased compared with control C57 mice (Figure 2C and 2D). Interestingly, there existed positive correlation between hippocampal Aβ1-40 and platelet Aβ1-40 in APP/PS1 mice, and the correlation degree was higher with the increase of age shown in Figure 3A (*r*=0.754, *p*=0.050; *r*=0.982, *p*=0.000). Similar outcomes were acquired between hippocampal Aβ1-42 and platelet Aβ1-42 in APP/PS1 mice shown in Figure 3B (*r*=0.856, *p*=0.014; *r*=0.882, *p*=0.009).

### Detection of APP cleaving enzyme

**Figure 4:**
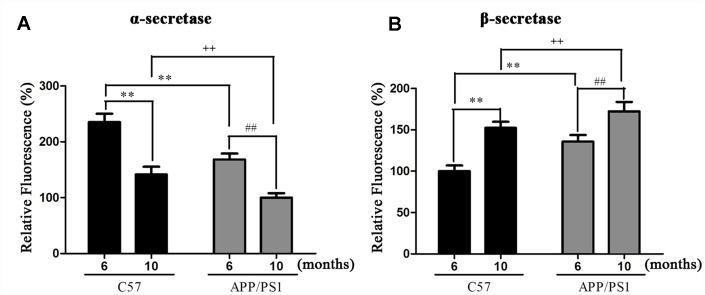
Activities of α- (**A**) and β-secretase (**B**) in platelets of APP/PS1 transgenic mice and C57 mice. ^**^P < 0.01 *vs.* C57 (6 months), ^##^*P* < 0.01 *vs.* APP/PS1 (6 months), ^++^*P* < 0.01 *vs.* C57 (10 months).

Detection of APP cleaving enzyme in the platelets. We further detected the expression of APP cleaving enzyme in platelets. Our results revealed that the expression of α-secretase significantly decreased and the β-secretase prominently increased in platelets of APP/PS1 mice compared with age-matched C57 group (Figure 4A and 4B).

### Measurement of oxidative stress markers

Measurement of oxidative stress markers in the platelets. MDA contents and activities of SOD, GSH-Px were used as oxidative biomarkers of aging models. As shown in Table 1, there was a dramatically overall group difference in MDA content, SOD activity and GSH-Px activity in platelet. When compared with age-matched C57 mice and young APP/PS1 mice, aged APP/PS1 mice showed a significant increase in the concentrations of MDA, accompany by a remarkable decrease in the activities of SOD and GSH-Px.

**Table 1:** The content of MDA and activities of SOD in platelets of APP/PS1 and C57 mice at various ages.

**Groups**	**C57**		**APP/PS1**	
	**6-mon**	**10-mon**	**6-mon**	**10-mon**
MDA (nM/mg)	4.45±0.46	6.90±0.69^*^	5.93±0.40^*^	8.79±0.61^#△^
SOD (U/mg)	103.65±7.03	79.68±5.46^*^	88.64±6.25^*^	60.34±6.21^#△^

^*^P < 0.01 *vs.* C57 (6 months), ^#^*P* < 0.01 *vs.* C57 (10 months), ^△^*P* < 0.01 *vs.* APP/PS1 (6 months).

### Protein expression of p-Akt, p-GSK3β, Bcl-2 and Cleaved caspase-3

**Figure 5:**
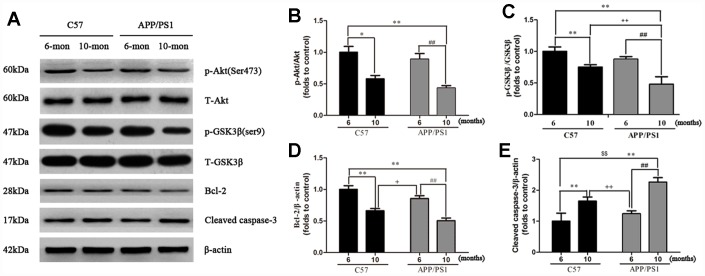
**Western blot analysis for p-Akt, p-GSK3β, Bcl-2 and Cleaved caspase-3 in the platelets of APP/PS1 transgenic mice and C57 mice.** Band intensities were quantified using Image J software. Each value was calculated on the basis of the data obtained from three independent experiment. Representative bar diagram showing quantitative results for relative levels of (**B**) p-Akt, (C) p-GSK3β, (**D**) Bcl-2, (**E**) Cleaved caspase-3. ^*^P < 0.05 *vs.* C57 (6 months), ^**^P < 0.01 *vs.* C57 (6 months), ^##^*P* < 0.01 *vs.* APP/PS1 (6 months), ^+^*P* < 0.05 *vs.* C57 (10 months), ^++^*P* < 0.01 *vs.* C57 (10 months).

Protein expression of p-Akt, p-GSK3β, Bcl-2 and Cleaved caspase-3 in the platelets. We attempted to determine whether PI3K/Akt/GSK3β pathway plays a role in regulating mitochondrial dysfunction in platelets. As shown in Figure 5, compared to the C57 group, the expressions of p-Akt (ser473), p-GSK3β (ser9) and Bcl-2 in platelets of APP/PS1 mice were significantly reduced compared with C57 mice; else, the expression of Cleaved caspase-3 was remarkable increased in platelets compared with C57 mice.

## DISCUSSION

Growing researches have been focused on the precise link between the intracellular Aβ cascade and the structure/function of mitochondria as early events in AD pathogenesis [3]. Mitochondrial dysfunction and oxidative stress have been observed in the mutant amyloid precursor protein (APP) transgenic mouse models of familial AD and in cell lines that express mutant APP [15]. Double transgenic mice share a spectrum of similar mitochondrial abnormalities such as mitochondrial loss, structural abnormalities, and functional distress with AD patients [16]. The mice show Aβ deposition at 6 months [17], so we used them at 6 and 10 months in the present study. We found that the inhibition of PI3K/Akt/GSK3β signaling pathway and regulations of apoptotic-associated proteins could be involved in Aβ-associated mitochondrial dysfunction of APP/PS1 mice and contribute to inhibition of APP and subsequent down-regulation of Aβ.

We, and others, have previously showed a strong association between mitochondrial Aβ levels and ATP damage in aging model mice [18, 19]. Mitochondria is not only the major site producing free radicals, but also the target that is susceptible to the attack of free radicals. Aging influences mitochondrial ATP synthesis, resulting in dramatic alterations of cerebral β-amyloid accumulation. Platelet is the most sensitive indicator of physical and chemical factors in peripheral blood [18], we therefore measured ATP levels in both hippocampi and platelets to evaluate functions of mitochondria. Our results indicate that aged APP/PS1 mice showed earlier ATP dysfunction than those of young APP/PS1 mice and age-matched C57 mice, which are consistent with previous reports that intracellular mitochondrial dysfunction is a vital cause of rapid aging in APP/PS1 mice [20]. These data support the existence of a mild deficiency in mitochondrial dysfunction before an age-associated ATP loss.

Aggregated amyloid-β (Aβ) accumulating in isolated mitochondria facilitates mitochondrial permeability transition opening, and leads to mitochondrial deficits including oxidative stress, energy deficiency and mitochondrial depolarization [21, 22]. Therefore, neuronal loss and memory deficits were frequently seen in Aβ treated cell models and Aβ overexpression animal models [7]. The full-length of APP was found in the brain and mitochondria of AD transgenic mouse [23] and phospho- APP is subsequently cleaved by the β-secretase and γ-secretase, thus results in the generation of the soluble APPβ fragment (sAPPβ) and Aβ. We showed that the expression levels of Aβ1-40 and Aβ1-42 were significantly increased in the hippocampi and platelets of APP/PS1 mice comparing to C57 wild-type mice at the same age. Interestingly, platelet and hippocampi show similar pattern in the levels of Aβ1-40 and Aβ1-42 in APP/PS1 mice. Furthermore, Aβ accumulation in APP/PS1 mice was increased by selectively inhibiting α-secretase activity and enhancement of β-secretase activity. Altogether, these data indicate that the decreased levels of ATP may reduce the abnormal interaction between Aβ and APP, leads to mitochondrial dysfunction, mitochondria supplying ATP to nerve terminals and boosts synaptic and cognitive function in AD.

Oxidative stress is another factor that induces mitochondrial dysfunction in the pathogenesis of AD [24]. Oxidative stress damage can be prevented by antioxidant defense system, which consists of non-enzymatic chain breaking antioxidants to scavenge of ROS production and antioxidase to eliminate ROS accumulation [24]. Therefore, we hypothesized there was a link between the increased ROS production and aging in platelets. So we measured the activities of lipid peroxidation, glutathione peroxidase and reductase which reflect the mitochondrial oxidative stress. As expected, aged APP/PS1 mice displayed the elevated levels of oxidative stress including the significant increase of MDA content and the dramatic decrease of SOD activities in platelets at an early age when compared with the young APP/PS1 mice and the age-matched C57 control mice. These results are consistent with earlier reports [25] that one possible mechanism promoting accelerated aging is the higher hyperoxidative status found in APP/PS1 strain compared with that in C57 mice. Together these data demonstrate that oxidative stress is an essential factor to cause the mitochondrial dysfunction in APP/PS1 mice.

**Figure 6:**
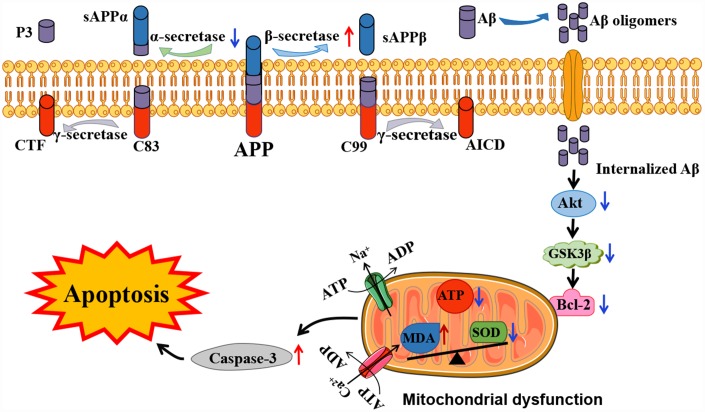
**Schematic representation of the potential mechanisms involved in Aβ-associated mitochondrial dysfunction in APP/PS1 mice. In the presence of Aβ, mitochondrial dysfunction has been shown to be associated with mitochondrial ATP production and cell signaling cascades, eventually leading to apoptosis.** The red and blue arrows visualize the increased or decreased processes of APP/PS1 mice proteins compared to the control C57 mice, respectively.

Previous studies revealed that activation of the phosphoinositide 3 kinase (PI3K)/Akt signaling pathway enhances the mitochondrial membrane potential and inhibits the production of reactive oxygen species, leading to regulate cellular processes and memory deficits [27, 28]. PI3K can activate downstream kinase Akt through phosphorylation at the ser473 residue and subsequently leading to phosphorylation of its downstream substrate GSK3β at ser9 residue, thus inhibits the activity of GSK3β [29]. In addition, Bcl-2, and cleaved-caspase-3 are important mediators of AD that are relevant to the regulation of mitochondrial apoptosis as the downstream targets of Akt [30, 31]. To fully dissect the role of mitochondrial dysfunction on Aβ-induced apoptosis in APP/PS1, we investigated the expression of p-Akt and its downstream targets, p-GSK3β, Bcl-2 and cleaved-caspase-3 in platelets. Consistent with previous studies [32], our findings showed decreased levels of pSer473-Akt and Bcl-2, and increased levels of pSer9-GSK3β and cleaved caspase-3 in the APP/PS1 group, when compared with the C57 group. Therefore, PI3K/Akt/GSK3β pathway was shown to involve in the dysfunction of mitochondria in APP/PS1 mice and contribute to inhibition of APP and subsequent down-regulation of Aβ (Figure 6).

In the present study, we showed that Aβ accumulation causes mitochondrial malfunction and oxidative stress in APP/PS1 mice, which reinforces Aβ deposition in a vicious cycle. These changes may due to the inhibition of PI3K/Akt/GSK3β signaling pathway and the down-regulation of anti-apoptotic proteins in platelets. Furthermore, abnormal metabolism of APP was shown in platelets in AD mice, and this provides potential biomarkers for early diagnosis of AD.

## MATERIALS AND METHODS

### Animals

**Figure 7:**
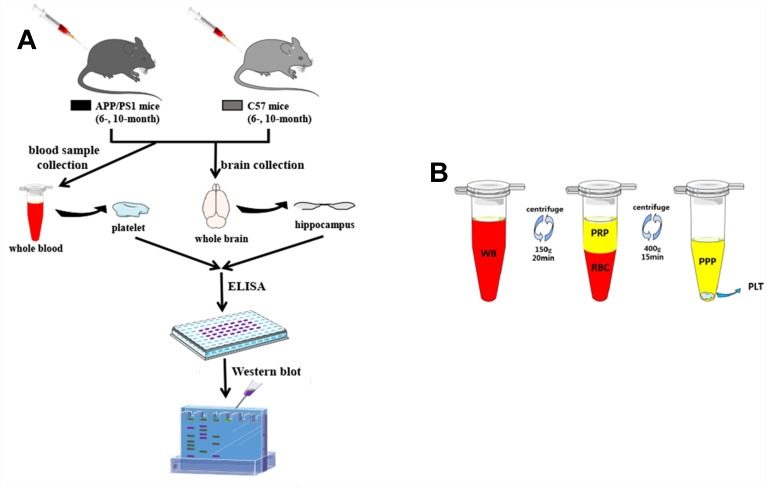
**Research protocol.** (**A**) An overview of the experimental scheme. (**B**) Graphic illustrating the platelet preparation from the tube-method protocol. The extraction of a small volume of whole blood (WB) was obtained from SAM-mice. After the single centrifugation at 150*g* for 20min, blood was fractionated into two layers: the bottom layer consists of red blood cells (RBC), and the top layer contains platelet rich plasma (PRP). The separated PRP was then centrifuged at 400*g* for 15min: the bottom consists of platelet pellets (PLT), and the top contains platelet poor plasma (PPP).

Experiments were carried out in APP/PS1 (APPswe/PSldE9) double-transgenic mice and wild-type C57BL/6J mice aged 6 and 10 months purchased from the Animal facility of Tianjin University of Traditional Chinese Medicine. The animals were housed in a light-dark cycle of 12 h at temperature 22±1°C in a humidity of 55±2% room with free access to tap food and water. The protocol employed here meets the guidelines for animal care and use of China and approved by the animal ethics committee of Sun Yat-sen University (Figure 7).

### Sample preparation for platelets and hippocampi

All mice were anesthetized with 10 g/L sodium pentobarbital, and blood samples were obtained by puncture of the right ventricle. Platelet-rich plasma (PRP) was achieved as shown in Figure 7. Briefly, appropriately 1 mL collected blood samples were drawn into EDTA-coated monoject tubes. After centrifuged at 150g for 20min at 4°C, the PRP was removed to another monoject tubes. Ultimately, platelet pellets were attained in the PRP by centrifuged at 400g for 15min.

After exsanguinated, mice were then immediately decapitated and brains were quickly for subsequent immersion in ice-cold homogenizing buffer for at least 5 min. Dissected hippocampi were homogenated by optimum ultrasonic wave and the supernatant was collected after centrifugation. The homogenate samples were subsequently snap-frozen in liquid nitrogen and stored at -80°C for the following biochemical analysis.

### ATP content of hippocampi and platelets

ATP contents in freshly prepared hippocampal and platelet homogenates were quantified using the ATP Bioluminescent Assay Kit (Biyutian, China). The reagents and reaction mixture were combined according to the supplied protocol using reversed-phase high-performance liquid chromatography (HPLC) with a calibrated ATP standard curve. Samples were monitored in triplicate. The ATP concentration was calculated as nmol ATP/mg protein, and then normalized by the control group (%).

### Enzyme-linked immuno sorbent assay (ELISA) for Aβ1-40 and Aβ1-42

Secreted concentrations of Aβ1-40 and Aβ1-42 in the hippocampi and platelets were quantitatively measured by commercially available Aβ ELISA kits (Invitrogen, USA) under the instruction of kit manual. Aβ levels were normalized to total protein content in the samples. The optical densities in each well were immediately determined by a microplate reader at 450nm, and Aβ1–40 and Aβ1–42 levels were expressed as pg/mg protein.

### α- and β-secretase assay

The α-Secretase Substrate II (Merck Millipore, Germany) and β-Secretase Activity Assay Kit (Biovision, USA) were performed to detect the activities of α- and β-secretase as described previously. The fluorescence intensities were measured with excitation wavelength at 340 nm and emission wavelength at 490 nm for α-secretase, and excitation wavelength at 350 nm and emission wavelength at 510 nm for β-secretase using a fluorescence microplate reader. The relative fluorescence unit (RFU) values were read by the standard curve and expressed as per mg of protein sample.

### Determination of oxidation

Levels of malonaldehyde (MDA) and activities of superoxide diamutase (SOD) in platelet were determined to examine the potential role of oxidant processes in mitochondrial dysfunction. The MDA levels and SOD activities were measured using corresponding commercial kits (Nanjing Jiancheng Bioengineering Institute, China). Protein measurement was established by Bicinchoninic Acid (BCA) assay using bovine serum albumin (BSA) as a standard.

### Western blot analysis

20 μg/lane protein lysate in platelets were loaded on SDS-PAGE gels and transferred onto polyvinylidene fluoride (PVDF) membranes. Following transfer, the membrane was preincubated with 5% nonfat milk blocking buffer and then sequentially incubated with primary antibodies against Akt (1:1000, CST), pSer473Akt (1:2000, CST), GSK3β (1:500, CST), pSer9GSK3β (1:2000, CST), Bcl-2 (1:500, CST), Cleaved caspase-3 (1:1000, CST) and β-actin (1:2000, CST) overnight at 4°C. Membranes were later incubated with horseradish peroxidase (HRP)-conjugated secondary anti-rabbit IgG (1:5000, CST) or anti-mouse IgG (1:5000, CST) for 2 h at room temperature. Subsequent visualization was carried out using the chemiluminescent HRP Substrate (Merck Millipore, Germany). Band intensities were quantified using Quantity One image analysis (Bio-Rad, USA), and then normalized to the intensity of β-actin.

### Statistical analysis

All statistical analyses were performed using SPSS 16.0 software. Values shown on the graphs were expressed as mean ± SD. One-way analysis of variance (One-way ANOVA) was performed to analyze the significance of the data followed by post hoc test. A pearson correlation was applied to examine the relationship between the hippocampal and platelet Aβ peptides. At least three independent experiments were carried out for in vitro study. Differences were considered significant at *P*<0.05.
